# NMDA Receptors in Glial Cells: Pending Questions

**DOI:** 10.2174/1570159X11311030002

**Published:** 2013-05

**Authors:** David Dzamba, Pavel Honsa, Miroslava Anderova

**Affiliations:** Department of Cellular Neurophysiology, Institute of Experimental Medicine AS CR, Prague, Czech Republic and Second Medical Faculty, Charles University, Prague, Czech Republic

**Keywords:** Astrocytes, ischemia, NG2 glia, NMDA receptors, oligodendrocyte progenitors, oligodendrocytes, polydendrocytes.

## Abstract

Glutamate receptors of the N-methyl-D-aspartate (NMDA) type are involved in many cognitive processes, including behavior, learning and synaptic plasticity. For a long time NMDA receptors were thought to be the privileged domain of neurons; however, discoveries of the last 25 years have demonstrated their active role in glial cells as well. Despite the large number of studies in the field, there are many unresolved questions connected with NMDA receptors in glia that are still a matter of debate. The main objective of this review is to shed light on these controversies by summarizing results from all relevant works concerning astrocytes, oligodendrocytes and polydendrocytes (also known as NG2 glial cells) in experimental animals, further extended by studies performed on human glia. The results are divided according to the study approach to enable a better comparison of how findings obtained at the mRNA level correspond with protein expression or functionality. Furthermore, special attention is focused on the NMDA receptor subunits present in the particular glial cell types, which give them special characteristics different from those of neurons – for example, the absence of Mg^2+^ block and decreased Ca^2+^ permeability. Since glial cells are implicated in important physiological and pathophysiological roles in the central nervous system (CNS), the last part of this review provides an overview of glial NMDA receptors with respect to ischemic brain injury.

## INTRODUCTION

### NMDA Receptors

NMDA receptors are named after their selective agonist N-methyl-D-aspartate and belong to the family of ionotropic glutamate receptors. The receptors are heterotetramers comprising a combination of GluN1, GluN2A-D and GluN3A-B subunits. Splice variants have been reported for most of the NMDA receptor subunits, but the best studied is the GluN1 subunit with 8 different isoforms [1]. The main agonists are glutamate and NMDA, with their binding site on GluN2 subunits, while the binding site for the co-agonists D-serine and glycine is located on GluN1 and GluN3 subunits. The most common composition of functional NMDA receptors involves two GluN1 and two GluN2 subunits, or two GluN1, one GluN2 and one GluN3 subunits. NMDA receptors composed of GluN1 and GluN3 subunits are also functional, but they lack the glutamate/NMDA binding site [2]. The large variety in possible NMDA receptor compositions leads to a large variation in their functional properties such as conductivity, deactivation time, sensitivity to Mg^2+^ and antagonist specificity. Furthermore, the composition of NMDA receptors differs among brain regions and also depends on the developmental stages [3]. Activated NMDA receptors are permeable to Ca^2+^, Na^+^ and K^+^; however, their permeability to certain ions is strongly dependent on the subunit composition, e.g., including the GluN3 subunit leads to decreased Mg^2+^ block and reduced Ca^2+^ and overall current permeability [4]. Since NMDA receptors are present on the majority of neurons and play a key role in mediating signal transduction in neuronal networks, they are the target of several drugs used for anaesthesia or the treatment of neurodegenerative diseases [5,6]. Interestingly, in the last 25 years a number of studies have emerged describing the presence of NMDA receptors also on glial cells [7]. 

### Glial Cells

It is not surprising that the first attempts to detect NMDA receptors on glial cells were performed on the most well-known and most abundant glial cell type – astrocytes. These cells consist of several subtypes based on the brain region and currently, researchers distinguish protoplasmic astrocytes in the grey matter, fibrillary astrocytes in the white matter, Bergmann glia in the cerebellum and Muller cells in the retina. The common property of these subtypes is their complex bushy morphology and the apparent larger size of their domains in larger–brained primates, possibly enabling the control of more synapses with fewer astrocytic somata [8]. The large surface area of astrocytes, their interconnection by gap junctions and their expression of leak potassium channels allow them to maintain K^+^ homeostasis in the CNS *via *K^+^ spatial buffering and thus to control neuronal activity [9]. In addition, astrocytes contribute to overall homeostasis in the neuronal tissue, including control of pH [10], the uptake of neurotransmitters [11], cerebral blood flow [12], the astrocyte-neuronal lactate shuttle [13], antioxidant function [14] and water transport [15]. An important feature of astrocytes is their ability to detect changes in their surroundings through many types of receptors. Beside a variety of metabotropic receptors, astrocytes also express ionotropic receptors for a large number of common neurotransmitters including glutamate [16]. Astrocytic endfeet which contain AMPA and NMDA receptors enwrap synaptic clefts and are thus able to modulate synaptic plasticity [7]. AMPA and NMDA receptors serve as sensors in this process; since they differ in their sensitivity to glutamate and their desensitization rate, they provide information about the duration and concentration of glutamate in the synaptic cleft. Moreover, astrocytes are able to release various chemical transmitters in a Ca^2+^-dependent manner [17]. Besides the classical types of astrocytes, there exist astrocyte-like adult neural stem cells (aNSC), which are localized in the SVZ and RMS and play an important role in the process of adult neurogenesis. Platel and co-authors described that neuroblasts use NMDA receptors to detect glutamate released in a Ca^2+^-dependent manner from aNSC [18]; however, the presence of NMDA receptors in aNSC has never been proven. 

Astrocytes also play an important role in many pathological processes. They react to injury by the increased expression of a variety of proteins, which causes their transformation into “reactive” astrocytes, a state with specific structural and functional characteristics. In CNS pathologies, reactive astrocytes represent a double-edged sword; on the one hand, they worsen the extent of injury due to the release of pro-apoptotic substances and Ca^2+^ to the surrounding healthy cells through gap-junctions, while on the other hand, they contribute to regenerative processes during the chronic phase of injury [19,20]. Unravelling the variety of newly expressed receptors and activated signalling pathways [21] that guide the activation of astrocytes may contribute to the development of therapies that could decrease the negative influence of astrocytes while enhancing their positive effect on the outcome of pathological processes.

When compared to the many complex functions of astrocytes, the role of oligodendrocytes in the CNS may appear to be less significant. However, this second most abundant glial cell population plays a crucial role in generating and maintaining myelin. By encapsulating neuronal axons with myelin, they enable the fast and efficient conduction of action potentials without any inappropriate increase in axonal diameter. Under pathological conditions oligodendrocytes, like neurons, are highly sensitive to damage by oxidative stress, trophic factor deprivation, the activation of apoptotic pathways and excitatory amino acids [22]. Such significant sensitivity to excitatory amino acids is probably caused by the expression and sustained activation of 2-amino-3-(3-hydroxy-5-methyl-isoxazol-4-yl) propanoic acid (AMPA), kainate or NMDA receptors, which initiate oligodendrocyte damage by cytoplasmic Ca^2+^ overload [23]. Describing the exact composition of these oligodendroglial receptors and discovering specific blockers could lead to the development of protective therapy in white matter pathologies without negative side effects on the neuronal population [24].

The third class of macroglia in the adult mammalian brain are cells expressing chondroitin sulphate proteoglycan NG2 [also known as oligodendrocyte precursor cells (OPCs),synantocytes or polydendrocytes]. These cells are widespread in the grey and white matter of the CNS; they comprise 8–9% of the total cell population in the white matter and 2–3% in the grey matter [25]. This glial cell population was first described more than 20 years ago [26]; however, only recently they have started to attract attention after the discovery of their special properties. Polydendrocytes are the most proliferative cells in the adult mammalian brain, as was shown in several studies that described that any given time, approximately half of them were in the interphase of the cell cycle [27-29]. A large number of studies sought to identify the reason for this extensive proliferative ability and found that a significant number of polydendrocytes change their phenotype and differentiate into other cell types. Polydendrocytes are able to divide symmetrically without retracting their processes or changing their morphology while giving rise to another polydendrocyte [27,30]; furthermore, these cells are also an important source of newly-derived postnatal oligodendrocytes [31]. Moreover, a number of studies have described the ability of these cells to differentiate not only into oligodendrocytes, but also into astrocytes and neuronal cells in the healthy [32] or injured CNS [33,34]. Another interesting feature of polydendrocytes is their ability to communicate with other cells. In contrast to astrocytes, which connect to each other by electrical synapses [35], polydendrocytes were shown to form classical chemical synapses with neighbouring neurons [36]; this may be an important communication channel that enables controlling polydendrocytic proliferation and/or differentiation. Polydendrocytes are equipped with a number of ionotropic ligand-gated receptors, predominantly AMPA or γ-aminobutyric acid (GABA) receptors; however, several studies also reported the surprising presence of receptors with properties similar to NMDA receptors. 

## ASTROCYTIC NMDA RECEPTORS

### Expression of NMDA Receptor Subunits in Astrocytes

In the last two decades there have been several attempts to detect subunits of NMDA receptors in astrocytes. In one of the first studies, Conti *et al*. used *in situ* hybridization together with immunocytochemistry to detect a marker of astrocytes – glial fibrillary acidic protein (GFAP) – and showed that in adult rat cortical slices virtually no astrocytes contained mRNA transcripts of the GluN1 subunit [37]. Other *in situ* hybridization studies in adult rat cerebellar Bergmann glial cells confirmed the presence of GluN2C and GluN2B mRNA transcripts [3,38] which; however, cannot form functional NMDA receptors without the GluN1 subunit. The absence of GluN1 transcripts in cultivated as well as acutely isolated astrocytes from 1-17 day-old mice was also confirmed by Affymetrix GeneChip Arrays [39]. Nevertheless, in mature acutely isolated cortical astrocytes, Cahoy and co-authors confirmed the presence of GluN2C transcripts, which were statistically highly enriched when compared to their numbers in neurons or oligodendrocytes. It is very interesting that in all astrocytes tested, a high level of GluN3A subunit transcripts was detected. In the study of Cahoy both the genes coding GluN2C and GluN3A subunits were statistically augmented in acutely isolated astrocytes when compared to cultured ones. In contrast to these findings, there are also studies showing the presence of the crucial GluN1 mRNA in astrocytes. In the first of these, reverse transcriptase polymerase chain reaction (RT-PCR) analysis of fluorescence-activated cell sorted astrocytes acutely isolated from the cortices of 2-week-old GFAP/EGFP transgenic mice revealed the presence of GluN1, GluN2B and GluN2C and the absence of GluN2A, GluN2D and GluN3 mRNA transcripts [40]. In another work studying astrocytes in primary cultures prepared from newborn mice, the authors showed the presence of GluN1 mRNA transcripts in these cells, which was significantly down-regulated after 2 weeks of cultivation [41]. On the other hand, the mRNA levels of GluN2A and GluN2B significantly increased with cultivation time for up to 4 weeks. Another very interesting result, especially with respect to human medicine, was obtained using end-point PCR results and showed that cultivated astrocytes isolated from both adult humans and human fetuses express the mRNA of all known NMDA receptor subunits, GluN1, GluN2A-D and GluN3A-B [42]. 

The first attempt to detect NMDA receptors in astrocytes on the protein level was performed in the rat visual cortex. GluN1 immunoreactivity in the astrocytic processes was detected by electron microscopy, especially in the deeper cortical layers starting at postnatal day 14. By postnatal day 30, the GluN1 immunoreactive profile in these layers became predominantly astroglial [43]. GluN1 and GluN2A/B immunoreactivity in adult rat cortical astrocytes was also confirmed by Conti and co-authors and was detected primarily in the astrocytic processes and occasionally in the cell bodies [44]. In astrocytic primary cultures prepared from the cortex of newborn mice, only a few astrocytes were GluN1- or GluN2B-positive, while the majority of them was negative [41]. The presence of NMDA receptor subunits seems to be brain region-specific, since in the CA1 region of the adult rat hippocampus, no GluN1 or GluN2A/B immunostaining was detected in astrocytes under physiological conditions [45,46]. In contrast to the hippocampus, the GluN1 protein was detected in adult rat astrocytes in the basolateral amygdala and the bed nucleus of the stria terminalis [47], the lateral and basal nuclei of the amygdala [48] and the nucleus locus coeruleus [49]. The results obtained in rat astrocytes are in accord with those reported from studies on human astrocytes. Conti *et al*. showed that the processes of human cortical astrocytes are immunopositive for GluN1, GluN2A and/or GluN2B subunits [50]. The study of Lee and colleagues confirmed the presence of all NMDA receptor subunits located on the membrane of cultivated astrocytes isolated from 16-19 week-old fetuses [42]. GluN1-positive cells were also found in cultured Muller glial cells isolated from the adult human retina, as confirmed by Western blots [51].

### NMDA-specific Current/Ca^2+^ Entry in Astrocytes 

There are a large number of functional studies providing insight into the astrocytic NMDA receptors enigma and clearly showing that astrocytic NMDA receptors are brain region-specific.

#### Cerebral Cortex

**Fig. (1):**
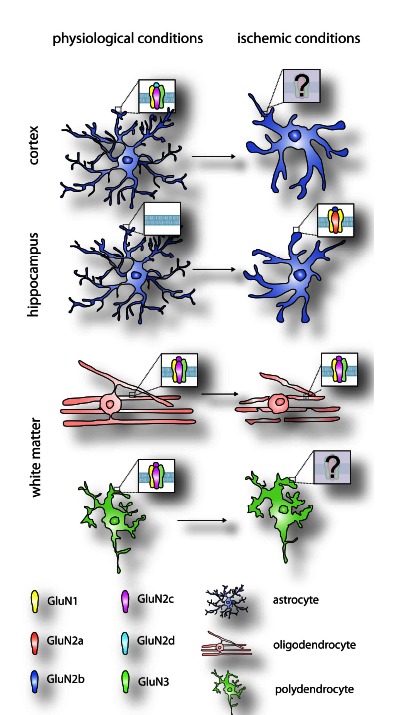
**The most probable composition of NMDA receptors in glial cells.** The most probable composition of NMDA receptors in astrocytes, oligodendrocytes and polydendrocytes in particular CNS regions under physiological and pathological conditions, based on published data predominantly from functional studies. Question marks indicate unknown NMDA receptor composition. Note the presumed absence of NMDA receptors in hippocampal astrocytes under physiological conditions.

In the cerebral cortex, the first attempts to detect NMDA receptors in astrocytes were performed on murine cultures prepared from neonatal animals. Even though glutamate, aspartate, kainate and several other neurotransmitters evoked electrophysiological responses, in none of these studies were NMDA-specific responses detected in response to NMDA application [52-55]. Similar results were obtained in calcium imaging experiments on cultured cerebral astrocytes [56-58]. The only exception was the study of Zhang and co-authors, in which the NMDA receptor antagonist (2R)-amino-5-phosphonopentanoate (D-AP5) diminished the Ca^2+^ response to glutamate [59]. Completely different results were obtained when cortical astrocytes were studied in slices from juvenile and adult mice. The application of NMDA triggered inward currents in most astrocytes, which were partially reduced by Cd^2+^ and almost completely and irreversibly blocked by the NMDA receptor antagonist dizocipline (MK-801) [40]. Since a cocktail containing tetrodotoxine (TTX), L-trans-pyrollidine-2,4-dicarboxylic acid (PDC), Cd^2+^ and the competitive AMPA/kainite receptor antagonist 6-cyano-7-nitroquinoxaline-2,3-dione (CNQX) reduced NMDA-evoked responses by ~45%, Shipke and co-authors suggested that most of the NMDA-induced currents are due to an indirect effect *via *neuronal glutamate release and glial glutamate uptake, but there is still a component indicating the presence of intrinsic NMDA receptors in astrocytes. Calcium imaging experiments showing NMDA-evoked Ca^2+^ elevations, especially in distal astrocytic processes, in the presence of the above-mentioned cocktail of inhibitors leads to the same conclusion. The application of 4 mM Mg^2+^ abolished the NMDA-induced current, and this blockage could be overcome only by depolarizing the cell membrane. Recently, two studies proved the presence of NMDA receptors on astrocytes by measuring the glial synaptic currents evoked by stimulating neurons. These currents were blocked or partially blocked by the NMDA antagonists MK-801, D-AP5, UBP141 and memantine, but not by ifenprodil [60,61] and they were maximal at 6 months of age, then decreased with increasing age [62]. These results were also verified by calcium imaging measurements that showed Ca^2+^ elevations after NMDA application, with a maximal amplitude reached between 3 and 6 months of age [62,63]. Similarly, NMDA-evoked currents were detected in cerebral astrocytes [64], however without blocking neuronal activity by TTX. The only work that cast doubt on the presence of NMDA receptors on cortical astrocytes *in situ* was published in 1997 by Pasti and co-authors [65], who reported that Ca^2+^ elevations were delayed 20-30 s following NMDA application, in contrast to the immediate neuronal responses reported by other authors. This might be due to the fact that in contrast to previous studies, Pasti and colleagues carried out their experiments on relatively young animals (5-day-old mice). The ultimate proof of the presence of functional NMDA receptors on cerebral astrocytes was provided by astrocytes acutely isolated using a vibrating ball technique, studies in which the cells were isolated from the possible influence of other surrounding cells. In these cells, NMDA-induced inward currents were potentiated by the addition of glycine and the responses were blocked by MK-801 or partially blocked by UBP141, while ifenprodil had no influence [61,63]. In contrast to the data of Shipke and co-authors, these astrocytes were insensitive to blockage by Mg^2+^. The NMDA receptor-mediated currents were maximal at 6 months, followed by a decline with increasing age [62]. NMDA application also induced Ca^2+^ responses in these acutely isolated cerebral astrocytes with lower amplitudes than those evoked by glutamate, and they were D-AP5 sensitive [61,63]. As for the NMDA receptor subunit composition, the lack of Mg^2+^ block together with lower Ca^2+^ permeability strongly suggest the presence of GluN3 subunits. Pharmacological studies suggest the incorporation of GluN2C/D subunits in astrocytic NMDA receptors. Therefore, the most probable composition of the NMDA receptors in cortical astrocytes seems to be tri-heteromeric, containing GluN1, GluN2C/D and GluN3 subunits [61] (Fig. **1**). For an overall summary of NMDA receptor subunits in astrocytes, see Table **1**. 

#### Hippocampus

The story of astrocytic NMDA receptors in the hippocampus seems to be much more complicated than in the cortex. The first experiments were performed on rat neonatal hippocampal cultures, and in contrast to glutamate, the application of NMDA or glycine did not evoke any Ca^2+^ responses in astrocytes [66]. This is in accord with another study performed on hippocampal astrocytes in cultures [46]. An interesting and so far unique result was obtained by Steinhauser and co-authors when they applied 5 mM NMDA to hippocampal astrocytes in slices prepared from juvenile mice, resulting in inward currents in 9 astrocytes, no response in 6 and outward currents in 7 cells tested. The measurements were performed without neuronal blocking, and similar results were obtained with 1 or 0.2 mM NMDA [67]. The hippocampal astrocytes in slices displayed an immediate NMDA-induced decrease in input resistance of 63%, which was significantly reduced by 43% in the presence of TTX [68]. However, the Ca^2+^ responses to NMDA application were delayed by at least 2 min in this study, raising a question about the cause of such a delay. In another work of Serrano and colleagues, a similar delay between the application of NMDA and the Ca^2+^ response of hippocampal astrocytes was observed with significantly reduced response amplitudes and even prolonged delays of the NMDA-evoked response in the presence of TTX [69]. In contrast, Porter and co-authors demonstrated that the Ca^2+^ responses of hippocampal astrocytes to NMDA are almost immediate, but the fact that TTX blocked 55% of these responses suggests that neuronal action potentials are also partially involved in triggering such increases in astrocytic intracellular Ca^2+^. When glutamate was applied to hippocampal astrocytes in the presence of TTX, the co-application of D-AP5 reduced the peak amplitude of the Ca^2+^ responses by 10% [70], which is; however, only an indirect indication that these astrocytes possessed functional NMDA receptors. On the other side of the spectrum of studies focusing on NMDA receptors in hippocampal astrocytes is the work of Shelton and co-authors, in which no Ca^2+^ increase was observed in response to NMDA application in the presence of TTX in any of 56 cells tested [71]. These ambivalent results might be due to the inherent complexity of the problem, especially when the neurons in the surrounding tissue have to be taken into account. Acutely isolated hippocampal astrocytes should help to overcome such a problem; however, NMDA complemented with glycine in Mg^2+^-free medium did not evoke any increases in membrane currents [72] or in the extent of Ca^2+^ elevation [73], indicating that acutely isolated hippocampal astrocytes do not possess any functional NMDA receptors under physiological conditions. On the other hand, the impact of the isolation procedure should be also considered. Employing papain for tissue dissociation might result in damaging or removing the receptors from astrocytic processes. Taken together, the existence of NMDA receptors in hippocampal astrocytes is even after more than two decades of research, still a matter of debate.

#### Cerebellum

NMDA receptors have also been studied in Bergman glia in the cerebellum. Neither NMDA nor aspartate elicited any current responses in cultivated astrocytes prepared from 8-day-old rats even in the presence of glycine or in Mg^2+^-free solution [74]. Nevertheless, the *in situ* study of Muller and co-authors showed that the application of NMDA induces an inward current in 30% of Bergmann glial cells, which was blocked by ketamine. In contrast to neurons, the presence of Mg^2+^ did not alter the amplitude of the inward currents. The NMDA-induced currents were also not affected by the presence of glycine, although the application of glycine itself induced a small inward current. In calcium imaging measurements performed simultaneously with electrophysiological measurements, no Ca^2+^ elevations were observed upon NMDA application even in cells responding to this application by an inward current. [75]. In another study, the application of NMDA induced Ca^2+^ responses in Bergmann glia in slices, but these were reversibly inhibited by TTX, suggesting that the Bergmann glia responded to a substance(s) released from neurons after firing action potentials [76]. 

#### Spinal Cord 

Cultured spinal cord astrocytes isolated from rat embryos produced only small Ca^2+ ^elevations after the application of NMDA, and in Ca^2+^-free solution NMDA application did not produce any Ca^2+^ responses at all [77]. Astrocytes in the spinal cord were also studied *in situ*. The application of NMDA induced a current in 95% of astrocytes with no sensitivity to Mg^2+^ or Ca^2+^-free extracellular solutions. These responses were observed in astrocytes in the spinal cords of very young animals, and the NMDA-induced current amplitude decreased with age to almost zero in 13-day-old rats [78]. Glial cells in the red nucleus of the brain stem *in situ* did not display any increase in membrane currents following the application of NMDA [79].

#### Studies on Human Astrocytes

Functional studies of NMDA receptors in human astrocytes have revealed their peculiar behavior. In cultivated astrocytes isolated from human cerebral white matter surrounding a brain tumor, the application of NMDA generated currents potentiated by glycine, inhibited by 19% in Ca^2+^-free extracellular solution and blocked by Mg^2+^. The currents were not sensitive to the NMDA receptor antagonists D-AP5 or kynurenic acid. Interestingly, a broad G-protein inhibitor, GDPβS, inhibited the NMDA-induced currents to 82% of the original levels, which suggests regulation by an unknown G-protein-coupled receptor [80,81]. Human Muller cells freshly isolated from adult human retinas showed NMDA-evoked currents sensitive to D-AP5 in 6 out of 8 cells tested. When these cells were cultivated, the NMDA-induced currents were gradually less frequently observed with no detectable NMDA responses after 2.5 months of cultivation. Reduction of the Ca^2+^ concentration in the bathing solution or pre-treatment with the Ca^2+^ chelator BAPTA-AM reduced the effect of NMDA on the inward currents, which suggests that the activation of NMDA receptors in human Muller cells leads to an influx of Ca^2+ ^[51].

### Other Functional Studies

Apart from electrophysiological and Ca^2+^ measurements, interesting results were also obtained by biocytin dialysis of a single astrocyte in mice hippocampal slices. This revealed that a 2 min bath application of NMDA resulted in a significant increase in the number of coupled astrocytes; however, when TTX was pre-applied, the increase in coupling was much smaller [68,69]. The authors suggested that the NMDA-induced increase in astrocyte coupling is indirect and involves the activation of the neuronal network. Studying cortical astrocytes in neuron/glia co-cultures prepared from neonatal rats, Kato and colleagues revealed that treatment with glutamate increases the levels of GFAP immunoreactivity. This effect was reversed by NMDA receptor antagonists. However, in purified astrocytic cultures, glutamate-induced astrocytic activation was not suppressed by antagonists of NMDA receptors [58]. Similar results were obtained in human retinal Muller cells in culture in which NMDA treatment stimulated the proliferation of these cells, suggesting the presence of functional NMDA receptors [82].

## NMDA RECEPTORS IN OLIGODENDROCYTES

Oligodendrocytes were thought to express predominantly non-NMDA glutamate ionotropic receptors, and initial reports also disclosed that oligodendrocytes do not respond to NMDA [83]. Nevertheless, oligodendrocyte NMDA receptors were shown to have an important role in controlling oligodendrocyte development [84], but also in oligodendroglial damage during pathological conditions.

### Expression of NMDA Receptor Subunits in Oligodendrocytes

The first evidence of NMDA receptors in oligodendrocytes was found *in vitro* in 1996, when Yoshioka and co-authors described the expression of NMDA receptor subunits using RT-PCR and Southern blotting in the rat oligodendroglial lineage CG-4 [85]. They found gene transcripts of GluN1 and GluN2D, and after the differentiation of CG-4 cells into oligodendrocyte-like cells, they found also transcripts of the GluN2B subunit. Despite the presence of the essential subunits for functional NMDA receptor formation, patch-clamp recordings revealed that NMDA application had no effect on this cell line [86]. Clear evidence of NMDA receptor expression in mature oligodendrocytes was provided by Salter and Fern, who demonstrated that cells of the oligodendrocyte lineage express different subunits of NMDA receptors [87]. In the white matter of CNP-GFP mice expressing green fluorescent protein (GFP) specifically in oligodendrocytes, RT-PCR analyses of NMDA receptor subunits disclosed the presence of mRNA coding the GluN1, GluN2A-D and GluN3A subunits. Moreover, mRNA quantification suggested that GluN1, GluN2C and GluN3A are the most abundant subunits in the optic nerve isolated from 7-13-day-old animals, while the levels of mRNA coding the GluN2B subunit were quite low and the GluN3B subunit was not present [87]. Similarly to previous reports, RT-PCR analyses of the adult rat optic nerve revealed the presence of mRNA coding the GluN1 subunit, namely three C terminal variants and two N terminal variants, as well as mRNA coding GluN2A-D and GluN3A [88]. Surprisingly, Burzomato and colleagues also detected mRNA coding the GluN3B subunit, a contrasting finding to that of Salter and Fern [87], a discrepancy that might originate from Salter and Fern’s use of the mouse optic nerve. Employing Affymetrix GeneChip Arrays, Cahoy and co-authors carried out gene expression profiling of glial cells and detected significant expression levels of only GluN3A transcripts in oligodendrocytes isolated from the forebrain of postnatal mice. Interestingly, other transcripts of NMDA receptor subunits were either not present or did not exceed the threshold level of significant expression [39]. 

The identification of oligodendroglial NMDA receptor subunits on the protein level and their localization were initially performed in optic nerves by Salter and Fern [87]. Using immunohistochemical analyses they found the clustered expression of the NMDA receptor subunit GluN1 along oligodendroglial processes, and they also observed GluN2A-D and GluN3A subunit expression with a high degree of co-localization with GluN1 and Glu2A subunits on oligodendrocyte processes. Concurrently, Micu and co-authors detected GluN1, GluN2 and GluN3 subunits appearing in discrete high densities in the myelin of larger axons in adult rat optic nerves [89]. Moreover, they were able to detect the GluN1 subunit on the rough endoplasmatic reticulum and Golgi membranes. Probing an isolated fraction of myelin from the adult rat optic nerve by immunoblotting with specific antibodies against NMDA receptor subunits provided evidence for GluN1/GluN3 but not GluN2/GluN3 complexes [90]. On the other hand, mature oligodendrocytes in the cerebellar white matter displayed strong immunoreactivity for GluN1, GluN2C and GluN3, while weaker labeling was observed for the GluN2A, B and D subunits [91]. Double-labeling revealed that the GluN1 and GluN2C subunits, and also the GluN3 and GluN2C subunits, co-localize in oligodendrocyte processes. Additionally, post-embedding electron microscopic immunohistochemistry showed that GluN1 subunits are present in the myelinating processes of adult cerebellar oligodendrocytes, in both the outer- and innermost membranes as well as within the myelin; their quantification disclosed that NMDA receptor density throughout the myelin is comparable to that observed in mossy fibre-granule cell synapses or parallel fibre-Purkinje cell synapses. These authors also noticed that the density of immunogold particles seemed to be higher in the outer membrane of the myelin, where the receptors presumably sense glutamate released from neighbouring cells [91]. Similarly, co-immunoprecipitation revealed interactions between the GluN1, GluN2C and GluN3A subunits in rat cerebellar oligodendrocytes [88]. Nonetheless, as for the myelination process, there are several studies that have shown the importance of NMDA receptors in immature oligodendrocytes, since even small Ca^2+^ signals can influence the stabilization/retraction of the myelinating projections from oligodendrocytes due to the small intracellular space [87,91,92]. However, De Biase and co-authors showed that this role of NMDA receptors in the myelination process can be taken over by AMPA receptors, since GluN1 subunit deletion from oligodendrocyte precursor cells led to the enhanced expression of AMPA receptors, but did not alter the formation of white matter tracts [93].

### Functional NMDA Receptors in Oligodendrocytes

Indirect evidence of functional NMDA receptors in oligodendrocytes came in 1993, when Matute and Miledi [94] described NMDA receptors in Xenopus leavis oocytes injected with mRNA from the corpus callosum. Supposing that oligodendrocytes represent the most abundant cell type in the corpus callosum, they suggested that in addition to AMPA/kainite receptors, these cells also express NMDA receptors. Using the whole-cell patch-clamp technique, NMDA-induced inward currents were detected in neurohypophysial O-2A cells, displaying properties similar to those of the neuronal NMDA receptor [95]. The NMDA-evoked currents were characterized by a range of activating NMDA concentrations from 5 to 100 µM, blockage by MK-801, and weak blockage by Mg^2+^. In addition, the authors showed that NMDA-evoked increases in intracellular Ca^2+^ are sensitive to the specific antagonist D-AP5 as well as to Ca^2+^-free solutions or high Mg^2+ ^[95]. In grey matter oligodendrocytes, direct evidence of functional NMDA receptors was given by Ziak and co-authors in spinal cord slices of young 5-13-day-old rats [78]. They described significantly higher NMDA-evoked currents in oligodendrocytes of the dorsal rather than the ventral horn and, moreover, an increase in NMDA-evoked currents during oligodendroglia maturation. Recently, in a few independent studies, functional NMDA receptors were clearly demonstrated in white matter oligodendrocytes in the cerebellum [91,96], corpus callosum [91] and adult rat optic nerve [89]. They showed that the NMDA-mediated responses were mostly restricted to the oligodendrocytic processes rather than to the cell somas and Karadottir and co-authors suggested that NMDA receptor expression might be a general feature of white matter oligodendrocytes [91]. NMDA-evoked currents recorded in oligodendrocytes of the corpus callosum and cerebellum were comparable in size to those evoked by AMPA or kainate, and based on pharmacological analyses of the NMDA-evoked currents, these authors proposed the presence of subunit combinations or even a mixture of NMDA receptors with different subunit compositions. Based on the pharmacological properties of the NMDA-evoked currents, their weak blockage by Mg^2+^ and immunohistochemical analyses revealing the co-localization of GluN1 and GluN2C subunits and also GluN3 and GluN2C subunits, they suggested that oligodendrocyte NMDA receptors comprise at least GluN1, GluN2C and GluN3 (Fig. **1**). Similarly, by comparing NMDA-evoked currents in cerebellar oligodendrocytes with those evoked in HEK cells transfected with cDNAs encoding recombinant receptors (GluN1/GluN2C/GluN3A or GluN1/GluN2C), Burzomato and co-authors clearly demonstrated that NMDA-evoked currents in oligodendrocytes reflect the activation of receptors containing GluN1, GluN2C and GluN3A [88].

Generally, NMDA receptors are typically activated by a combination of glutamate and glycine; however, Piňa-Crespo and co-authors recently described excitatory responses in optic nerve myelin, gated by glycine alone and mediated by GluN1/GluN3 NMDA receptor subunits. They suggested that D-AP5-insensitive/2,3-dihydroxy-6-nitro-7-sulfamoyl-benzo[f]quinoxaline-2,3-dione (NBQX)-sensitive GluN1/GluN3 receptors seem to be restricted to myelin [90], while D-AP5 sensitive GluN2-containing receptors are more widely distributed [87,89,91]. For an overall summary of NMDA receptor subunits in oligodendrocytes, see Table **1**.

## NMDA RECEPTORS IN POLYDENDROCYTES

### Expression of NMDA Receptor Subunits in Polydendrocytes

One of the first studies describing the presence of NMDA receptors on the mRNA level was performed on oligodendrocyte precursors that were isolated from neonatal rats and kept under* in vitro *conditions by Wang and co-authors [95]. mRNA coding the GluN1 subunit was clearly detected using *in situ* hybridization and RT-PCR; however, their analyses failed to prove the presence of GluN2A-D subunit transcripts. Strikingly, the number of studies that report the expression of NMDA receptor subunits on the mRNA level in polydendrocytes* in situ* is smaller than the numerous functional studies that report the same finding; nevertheless, Cahoy and colleagues [39] recently showed the strong expression of mRNA coding the GluN3A subunit in polydendrocytes acutely isolated from postnatal mice. Similarly to oligodendrocytes, they did not detect mRNA encoding other NMDA receptor subunits. 

Another important step towards confirming the presence of NMDA receptors subunits in polydendrocytes was done on the protein level by Wang and co-authors. Their pioneer study was performed *in vitro* on neonatal rat oligodendrocyte precursors, in which the GluN1 subunit was detected on process-bearing cells [95]. Recently, two studies confirmed the expression of several NMDA receptor subunits in polydendrocytes *in situ*. Manning and colleagues [97] detected GluN1 subunits, which are required for assembling a functional NMDA receptor, on O4-positive polydendrocytes in the white matter of postnatal rats and in developing human tissue. The second study employed the transgenic mouse strain NG2-dsRed, where polydendrocytes labelled with red fluorescent protein in the 15-day-old cortex expressed, besides AMPA receptors, also GluN2A and GluN2C subunits in the cell bodies as well as in the primary processes. Some of these NMDA receptors co-localized with synaptophysin, an indicator of synaptic contacts [98]. 

### Functional NMDA Receptors in Polydendrocytes

In contrast to the low number of studies describing the presence of NMDA receptors in polydendrocytes at the mRNA or protein level, numerous references exist in the literature that report functional properties indicating the presence of NMDA receptors on this glial cell type. Wang and co-authors [95] detected for the first time the capability of cultivated oligodendrocyte precursors to respond to an application of NMDA using the whole cell patch-clamp recordings *in vitro*. These cells displayed an inward current in the range of 20-100 pA that slowly inactivated upon prolonged application of NMDA and was inhibited by D-AP5, a selective competitive NMDA receptor antagonist. Interestingly, the responses were very similar to those measured in neurons, based on a significant Mg^2+^ block and strong Ca^2+^ permeability, as revealed by calcium imaging. Another important advance in identifying functional NMDA receptors was done on white matter polydendrocytes *in situ* in 1-2-week-old rats. This type of cell responded to the application of glutamate by an inward current that was partially blocked by D-AP5 or MK-801 and fully blocked by NBQX, thus demonstrating the significant contribution of NMDA receptors to glutamate-evoked currents in addition to the strong activation of AMPA receptors [91]. Moreover, these data point to NMDA receptors of unusual subunit composition (probably GluN1, GluN2C and GluN3 subunits, Fig. **1**) with a weak Mg^2+^ block that are, however, more prominent in pre-myelinating and mature oligodendrocytes. Using another NMDA receptor antagonist, (+/-)-3-(2-carboxypiperazin-4-yl)-propyl-1-phosphonic acid (D,L-CPP), revealed that more than 60% of polydendrocytes in the white matter of adult Ng2-dsRed mice produce currents in response to NMDA application that were sensitive to this NMDA antagonist [99]. In contrast to previous reports, Ziskin and colleagues showed that NMDA currents in polydendrocytes are also blocked by Mg^2+^ at their resting potential, indicating that substantial depolarization would be required to allow Ca^2+^ influx through these receptors. It is worth noting that another study, which examined the NMDA-evoked currents in polydendrocytes from the cerebellum of 7-day-old rats, distinguished two different groups of polydendrocytes with distinct responses to the application of NMDA. One group comprised cells with non-detectable voltage-activated sodium TTX-sensitive currents that responded only weakly to the application of NMDA, while the second group consisted of polydendrocytes with large Na^+^ currents and NMDA-evoked inward currents that were significantly higher than those observed in the first group [100]. The optic nerve was another region of the CNS where the expression of polydendrocytic NMDA receptors was examined. In this region approximately 22% of measured cells showed an increased intracellular concentration of Ca^2+^, based on Fura-2 calcium imaging, and these responses were sensitive to D-AP5 antagonist [98]. One of the most conclusive approaches was used by De Biase and co-authors [93], who generated triple transgenic mice (called PDGFαR–CreER;NR1^flox/flox^;Z/EG) that enabled conditional tamoxifen-inducible knock-out of obligatory GluN1 subunits in PDGFαR-expressing polydendrocytes. The application of tamoxifen resulted in the genetic deletion of GluN1 subunits of the NMDA receptor specifically in polydendrocytes, and subsequent electrophysiological measurements in the white matter of 30-day-old mice showed the disappearance of D,L-CPP-sensitive NMDA currents after this conditional knock-out. For an overall summary of NMDA receptor subunits in polydendrocytes, see Table **1**. Nevertheless, the aim of this study was to reveal the function of NMDA receptors in the oligodendrocyte lineage, and it was clearly demonstrated that NMDA receptor ablation did not affect any physiological or morphological properties of polydendrocytes or their synaptic connections, survival or differentiation. These findings raise questions about the function and significance of NMDA receptors in polydendrocytes in the mammalian brain. 

As shown above, a number of convincing indications exist on mRNA, protein and functional levels that prove the presence and functionality of NMDA receptors on polydendrocytes in a number of CNS regions during several developmental stages as well as in adulthood. Nevertheless, it seems that NMDA receptors are not the major mediators of glutamate signals from surrounding cells. This role is evidently conveyed by Ca^2+^ permeable AMPA receptors, which respond to this excitatory neurotransmitter with stronger responses while NMDA receptors probably serve as a feedback control of AMPA receptor function [93]. However, some properties of polydendrocytic NMDA receptors are striking. Although there are several contradictory results in the literature, it seems that their subunit composition differs from that found in the majority of neurons, which could result in different functional properties compared to neuronal NMDA receptors, such as a lack of Mg^2+ ^block at resting membrane potential or decreased permeability for Ca^2+^. These unusual properties of NMDA receptors in polydendrocytes may be one of the reasons for their being overlooked for an extended period in the past.

## GLIAL NMDA RECEPTORS IN THE PATHOLOGY OF ISCHEMIA 

Excessive activation of ionotropic glutamate receptors occurring under ischemic conditions is harmful to neurons but also to glial cells. There have been two studies examining NMDA receptors in astrocytes after an ischemic insult, both in the hippocampus. Transient forebrain ischemia in rats resulted in the appearance of GluN2A/B but not GluN1 immunolabeling in cells with the characteristic morphological features of astrocytes, with the maximum expression levels appearing several weeks after ischemia [45]. The second study of Krebs and colleagues showed that transient forebrain ischemia resulted in the increased expression of both GluN1 and GluN2B subunits in GFAP-positive hippocampal astrocytes, reaching a maximum 14 to 28 days after ischemia [46] (Fig. **1**). Hippocampal astrocytes isolated from 2-4-day-old rats and cultivated in the presence of neurons were not immunopositive for any NMDA subunit, but after anoxia, which led to neuronal death, the GluN1 and GluN2B subunits became expressed in the remaining GFAP-positive glial cells. It is worth noting that when these astrocytes were cultivated in the absence of neurons, they were not positive for either GluN1 or GluN2B prior to or after anoxia. These authors further demonstrated that astrocytes co-cultivated with neurons 3 days after anoxia responded to the application of NMDA by an oscillatory increase in intracellular Ca^2+^ levels, which was reversibly blocked by D-AP5. The authors speculated that the expression of NMDA receptors in cultivated hippocampal astrocytes is probably a response related to the accompanying neuronal death. The final proof of ischemia-induced NMDA receptor appearance in hippocampal astrocytes was shown in cells acutely isolated from brains 20 days after transient forebrain ischemia, in which NMDA application elicited an increase in intracellular Ca^2+ ^levels that was partially blocked by ifenprodil and fully blocked by D-AP5 [46]. Nevertheless, the impact of an ischemic insult on NMDA receptors in other brain regions has yet not been studied. 

Among glia, oligodendrocytes are the most sensitive cells to excitotoxicity mediated by glutamate. Ca^2+^ entry, which is mediated by glutamate receptors, ultimately leads to oligodendroglial cell death *via *Ca^2+ ^accumulation in the mitochondria, their depolarization and the release of proapoptotic factors [5]. Despite the fact that AMPA/kainate receptors were described as the major mediators of excitotoxic death in mature oligodendrocytes due to GluA4 expression, which is associated with the high Ca^2+^ permeability of AMPA/kainate receptors [101-105], a large number of studies have emphasized the role of NMDA receptors in oligodendroglial pathophysiology following ischemic injury; for a review see [23]. Nevertheless, the mechanisms that are triggered by NMDA receptor overactivation and that lead to oligodendroglial damage have not yet been elucidated. In 2005 Salter and Fern reported that in oxygen-glucose deprived optic nerves, the activation of NMDA receptors results in the fast Ca^2+^-dependent detachment and disintegration of oligodendroglial processes in the white matter of CNP-GFP mice and that blocking the NMDA receptors prevents injury to oligodendrocytic processes [87]. They also found the differential expression of NMDA and AMPA/kainate receptors, which explains the early damage of myelin sheaths due to NMDA receptor activation, while AMPA/kainate receptor activation mediates cytotoxicity. Such detachment/disintegration of oligodendroglial processes was observed at an age corresponding to the onset of myelination as well as at the end of this process. Using an *in vitro* model of chemical ischemia, Micu and co-authors demonstrated that Ca^2+^ increases in myelin in adult rat optic nerves are abolished by NMDA antagonists, such as MK-801 or D-AP5, while those evoked in cell bodies are completely blocked by the AMPA/kainate receptor antagonist NBQX [89,106]. Additionally, it has been shown that ischemia leads to the development of an inward current in oligodendrocytes of the cerebellar cortex [91], corpus callosum and optic nerve [107], which is partially blocked by memantine or MK-801 and thus carried by NMDA receptors. NMDA receptor blockade with memantine was shown to attenuate white matter injury in a rat model of periventricular leukomalacia, a white matter injury resulting from hypoxia-ischemia in premature infants [97,108]. Although the protective effects of NMDA receptor antagonists were demonstrated *in vitro*, *in situ *as well as* in vivo* [87,89,91,97,106,107], the application of such antagonists might be somewhat problematic in human patients due to the significant side-effects of NMDA antagonists, such as interference with synaptic function. Nevertheless, all of these results show that inhibiting NMDA receptors alone is sufficient to protect against white matter injury and that the use of NMDA antagonists might be considered for clinical applications. However, the development of new selective agonists or antagonists should rather focus on the unusual subunit composition found in myelin/white matter oligodendroglia. 

Despite increasing interest in polydendrocytes, the impact of ischemia on NMDA receptors in these cells has not yet been studied. 

## THE ROLE OF GLIAL NMDA RECEPTORS IN CELL DEATH

Besides the most common type of CNS injury, which is the above-described ischemia, NMDA receptors play an important role in many other CNS pathologies such as traumatic brain injury, multiple sclerosis [23], as well as certain neurodegenerative diseases, such as Huntington’s [109]. In general, excessive glutamate release in the CNS can lead to the necrotic or apoptotic cell death of neurons as well as glial cells, and which of these two pathways prevails, depends mostly on the severity of the insult. As mentioned above, severe insults lead to necrosis due to excessive Ca^2+^ influx through NMDA receptors, which is taken up by mitochondria and causes an initial depolarization of the mitochondria. Overloading mitochondria with Ca^2+ ^causes necrotic cell death and might induce the production of reactive oxygen species that damage either mitochondrial respiratory processes or impair Ca^2+^ extrusion and membrane potential maintenance. The substantial depletion of cytosolic ATP levels caused by the reversal of mitochondrial ATP synthase in depolarized mitochondria further limits the ability of cells to regulate their intracellular Ca^2+^ levels [110]. On the other hand, apoptotic cell death occurs if the NMDA receptor-dependent insult is less intensive, mitochondrial depolarization is incomplete and intracellular ATP levels are sufficient to support the active processes associated with apoptosis. The mechanisms underlying NMDA receptor-dependent apoptosis are not certain, but are likely to involve the release of apoptotic factors – such as cytochrome c – from mitochondria and the activation of stress-induced pathways such as that of p38 MAP kinase or c-Jun N-terminal kinase [110].

Finally, glutamatergic signaling is not mediated only by NMDA currents. Many other glutamate receptors and transporters exist in glial cells. Simultaneously with NMDA receptor activation, glutamate binds and activates other glutamate receptors (AMPA, kainate) and transporters, including eight metabotropic glutamate receptor subtypes and the excitatory amino acid transporters 1,2 [7]. The activation of each of these receptors or transporters has the capacity to initiate cells death. Extensive studies in recent years have demonstrated that NMDA receptor subunits are capable of binding PDZ-domains, which not only ensures their proper targeting but may also play a critical role in coupling NMDA receptors to intracellular proteins and signaling enzymes [111-113]. This tight organization may thus facilitate the individual toxic signal mechanisms downstream of the receptors during glutamate over-activity. Evidence exists showing that the downstream signals of NMDA receptors activate proteins such as nitric oxide synthase, an enzyme that produces superoxide; the resultant oxidative stress participates in cell death. Another downstream activated signaling molecule is PARP-1, which plays a crucial role in apoptotic cell death [114].

Before the discovery of NMDA receptors in glial cells, a theory existed that excitotoxic damage of glial cells (predominantly oligodendrocytes) was related to the over-activation of AMPA and kainate receptors [115]. This theory was controversial because of the rapid desensitization of AMPA receptors, which inactivates them when exposed to glutamate for a long time. The discovery of NMDA receptors in glial cells made them prime suspects for such excitotoxicity. Based on glial-specific NMDA receptor subunit composition, these receptors have different pharmacokinetics, without Mg^2+^ block and lower Ca^2+^ permeability, when compared to neuronal NMDA receptors [7]. Although the overall Ca^2+^ influx through glial NMDA receptors could be minor under physiological conditions, the absence of Mg^2+^ block and a hyperpolarized glial resting membrane potential compared to neurons would provide for a larger and significant Ca^2+^ influx under pathologic conditions as a consequence of excessive extracellular glutamate concentration. 

## CONCLUSION

In summary, it seems that astrocytes, oligodendrocytes as well as polydendrocytes express NMDA receptors of quite similar composition, mainly containing the GluN1 and GluN2 subunits, but more importantly, the unusual GluN3A subunit as well (Fig. **1**). The co-assembly of these subunits within glial NMDA receptors markedly alters their properties, resulting in a weak or even absent sensitivity to Mg^2+^; as a result, glial NMDA receptors may be potentially active at the resting membrane potential, which is in contrast to neurons. Nevertheless, many published and surely many more unpublished studies have failed to prove the expression of NMDA receptors on glial cells, a phenomenon that could, however, be connected to the clear regional specificity in the expression of NMDA receptors on glial cells in the CNS and moreover, also to their probable age-dependent expression. It should also be taken into account that before discovering the composition and properties of NMDA receptors on glial cells, many studies were considered negative due to the expectation of observing NMDA-specific responses typical of neurons, which are not, in fact, characteristic of glial cells. Under physiological conditions, astroglial NMDA receptors were shown to be involved in neuronal-glial signalling [7]; nevertheless, the role of NMDA receptors in oligodendrocytes and polydendrocytes needs to be elucidated. On the other hand, the pathogenic potential of NMDA receptors was clearly demonstrated in oligodendrocytes during ischemic injury, while the role of astrocytic and polydendrocytic NMDA receptors in CNS pathologies such as ischemia remains unknown. NMDA-mediated Ca^2+^ entry might be one of the key players triggering polydendrocyte migration, proliferation and/or differentiation and may also contribute to the onset of events initiating the development of reactive astrocytes. All of the processes connected with NMDA receptors in glial cells described in this review, including their role in ischemia and the myelination process, naturally bring promising pharmacological perspectives [116,117]. There were several attempts to utilize antagonists of NMDA receptors in clinical trials; however, most of them were high-affinity antagonists that impede normal brain activity and lead to intolerable side effects. The exceptions are memantine derivatives, since these are open-channel uncompetitive antagonists with a relatively fast “off rate”, parameters that do not impede physiological neurotransmission, thus making them clinically tolerable [6,97,118-121]. Another reason why many clinical trials with NMDA receptor antagonists were unsuccessful is, in our opinion, their strong focus on neuronal NMDA receptors while neglecting these receptors in glial cells due to the relatively recent discoveries in this field. Nonetheless, thanks to the unusual composition of glial NMDA receptors (especially GluN2C and GluN3A subunits), we believe that new antagonists specific for glial NMDA receptors will emerge soon and will enable selective targeted intervention with weaker/fewer side effects. Although many questions about NMDA receptors in the human brain have been answered, numerous questions are still pending. Future research should thus unravel the exact role of glial NMDA receptors in CNS physiology/pathology since this will significantly contribute to the future treatment of many serious brain pathologies.

## Figures and Tables

**Table 1.** NMDA Receptor Subunits in Murine Glial Cells Under Physiological Conditions

	Level	Brain Area	NMDA Receptor Subunits							Reference
			GluN1	GluN2A	GluN2B	GluN2C	GluN2D	GluN3A	GluN3B	
astrocytes	mRNA	cortex	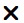							Conti *et al*., 1994
			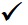		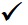	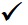				Schipke *et al*., 2001
			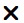			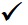		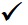		Cahoy *et al*., 2008
		cerebellum				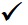				Akazawa *et al*., 1994
					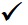					Luque *et al*., 1995
	protein	cortex	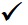							Aoki *et al*., 1994
			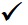	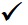	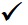					Conti *et al*., 1996
		hippocampus	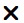	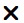	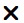					Gottlieb *et al*., 1997
			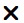		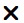					Krebs *et al*., 2003
		amygdala	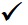							Gracy *et al*., 1995
			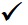							Farb *et al*., 1995
		nucleus locus coeruleus	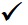							Bockstaele *et al*., 1996
	function	cortex	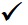			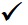	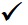	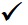	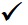	Palygin *et al*., 2010, 2011
oligodendrocytes	mRNA	optic nerve	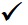			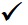		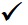		Salter and Fern, 2005
			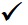	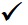	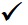	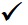	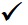	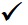	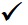	Burzomato *et al*., 2010
		forebrain	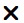			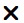		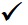		Cahoy *et al*., 2008
	protein	optic nerve	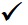	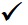	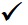	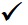	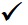	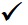		Salter and Fern, 2005
			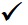	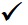	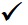	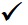	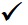	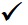	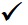	Micu *et al*., 2006
			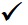					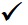	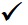	Piňa-Crespo *et al*., 2010
		cerebellar white matter	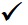			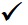		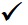	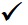	Karadottir *et al*., 2005
			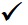			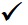		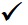		Burzomato *et al*., 2010
	function	white matter	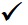			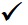		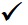	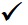	Karadottir *et al*., 2005
		cerebellar white matter	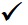			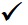		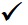		Burzomato *et al*., 2010
		optic nerve	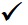					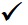	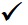	Piňa-Crespo *et al*., 2010
polydendrocytes	mRNA	forebrain				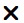		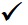		Cahoy *et al*., 2008
	protein	white matter	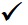							Manning *et al*., 2009
		cortex		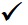		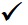				Hamilton *et al*., 2010
	function	white matter	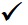			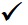		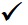	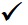	Karadottir *et al*., 2005
			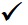							De Biase *et al*., 2011

The data were obtained from studies performed *in situ* and on freshly isolated glial cells, while those obtained *in vitro *were not included due to the possible influence of the cultivation process.
